# Standardised Outcomes in Nephrology – Chronic Kidney Disease (SONG-CKD): a protocol for establishing a core outcome set for adults with chronic kidney disease who do not require kidney replacement therapy

**DOI:** 10.1186/s13063-021-05574-1

**Published:** 2021-09-09

**Authors:** Nicole Evangelidis, Benedicte Sautenet, Magdalena Madero, Allison Tong, Gloria Ashuntantang, Laura Cortes Sanabria, Ian H. de Boer, Samuel Fung, Daniel Gallego, Andrew S. Levey, Adeera Levin, Eduardo Lorca, Ikechi G. Okpechi, Patrick Rossignol, Laura Sola, Tim Usherwood, David C. Wheeler, Yeoungjee Cho, Martin Howell, Chandana Guha, Nicole Scholes-Robertson, Katherine Widders, Andrea Matus Gonzalez, Armando Teixeira-Pinto, Andrea K. Viecelli, Amelie Bernier-Jean, Samaya Anumudu, Louese Dunn, Martin Wilkie, Jonathan C. Craig, on behalf of the SONG-CKD Investigators

**Affiliations:** 1 Sydney School of Public Health, The University of Sydney, Sydney, Australia; 2 Centre for Kidney Research, The Children’s Hospital at Westmead, Westmead, Sydney, Australia; 3 Department of Nephrology, CHU Tours, INSERM SPHERE U1246, University of Tours, University of Nantes, Tours, France; 4 Division of Nephrology, Department of Medicine, Instituto Nacional de Cardiología Ignacio Chávez, Mexico City, Mexico; 5 Department of Internal Medicine and Specialties, Faculty of Medicine and Biomedical Sciences, University of Yaounde I, Yaounde, Cameroon; 6 Unidad de Investigación Médica en Enfermedades Renales, Hospital de Especialidades, CMNO, IMSS, Guadalajara, Mexico; 7 Department of Medicine, Kidney Research Institute, University of Washington, Seattle, Washington USA; 8 Division of Nephrology, Department of Medicine & Geriatrics, Princess Margaret Hospital, Hong Kong, Hong Kong; 9 Federacion Nacional ALCER (Spanish Kidney Patient’s Federation), Madrid, Spain; 10 Division of Nephrology, Tufts Medical Center, Boston, MA USA; 11 Division of Nephrology, University of British Columbia, Vancouver, Canada; 12 Department of Nephrology, Hospital Salvador, Santiago, Chile; 13 Division of Nephrology and Hypertension, University of Cape Town, Cape Town, South Africa; 14 Department of Medicine, University of Alberta, Edmonton, Alberta Canada; 15 Université de Lorraine, Inserm, Centre d’Investigations Clinique 1433 and Inserm U1116; CHRU Nancy; F-CRIN INI-CRCT, Nancy, France; 16 Dialysis Unit, CASMU-IAMPP, Montevideo, Uruguay; 17 The University of Sydney, Westmead Clinical School, Westmead, NSW Australia; 18 The George Institute for Global Health, University of New South Wales, Sydney, NSW Australia; 19 Centre for Nephrology, UCL Medical School, London, UK; 20 Department of Nephrology, Princess Alexandra Hospital, Brisbane, QLD Australia; 21 Australasian Kidney Trials Network, Centre for Health Services Research, University of Queensland, Brisbane, QLD Australia; 22 Translational Research Institute, Brisbane, QLD Australia; 23 Selzman Institute for Kidney Health, Section of Nephrology, Baylor College of Medicine, Houston, TX USA; 24 Sheffield Kidney Institute, Sheffield Teaching Hospitals NHS Foundation Trust, Sheffield, UK; 25 College of Medicine and Public Health, Flinders University, Adelaide, Australia

**Keywords:** Core outcome set, Outcomes research, Patient-centred outcomes, Clinical trials, Chronic kidney disease

## Abstract

**Background:**

Globally, over 1.2 million people die from chronic kidney disease (CKD) every year. Patients with CKD are up to 10 times more likely to die prematurely than progress to kidney failure requiring kidney replacement therapy. The burden of symptoms and impaired quality of life in CKD may be compounded by comorbidities and treatment side effects. However, patient-important outcomes remain inconsistently and infrequently reported in trials in patients with CKD, which can limit evidence-informed decision-making. The Standardised Outcomes in Nephrology – Chronic Kidney Disease (SONG-CKD) aims to establish a consensus-based core outcome set for trials in patients with CKD not yet requiring kidney replacement therapy to ensure outcomes of relevance to patients, caregivers and health professionals are consistently reported in trials.

**Methods:**

SONG-CKD involves four phases: *a systematic review* to identify outcomes (domains and measures) that have been reported in randomised controlled trials involving adults with CKD who do not require kidney replacement therapy; *stakeholder key informant interviews* with health professionals involved in the care of adults with CKD to ascertain their views on establishing core outcomes in CKD; an *international two-round online Delphi survey* with patients, caregivers, clinicians, researchers, policy makers and industry representatives to obtain consensus on critically important outcome domains; and *stakeholder consensus workshops* to review and finalise the set of core outcome domains for trials in CKD.

**Discussion:**

Establishing a core outcome set to be reported in trials in patients with CKD will enhance the relevance, transparency and impact of research to improve the lives of people with CKD.

**Trial registration:**

Not applicable. This study is registered with the Core Outcome Measures in Effectiveness Trials (COMET) database: http://www.comet-initiative.org/Studies/Details/1653.

## Background

Globally, over 1.2 million people die from chronic kidney disease (CKD) every year and this is projected to reach at least 2.2 million by 2040 [1]. The prevalence of CKD also continues to rise, with the highest rates in low- to middle-income countries [2]. Patients with CKD are up to 10 times more likely to die prematurely than progress to kidney failure requiring kidney replacement therapy, largely due to cardiovascular disease [3, 4]. The burden of symptoms and impaired quality of life in CKD may be compounded by comorbidities, including diabetes and cardiovascular disease [5, 6], complications, medication side-effects and having to confront the potential need for dialysis or transplant.

Informed decision-making remains limited because the outcomes of importance to patients with CKD who do not yet require kidney replacement therapy are not consistently reported in clinical trials. Fatigue, life participation, anxiety and depression are of priority to patients with CKD and their caregivers [7]; however, these are not frequently measured and in particular, patient-reported outcomes are often omitted from trials. Instead, surrogate biochemical outcomes are more frequently reported in trials in CKD because of feasibility [8]. Systematic reviews have repeatedly shown that the outcomes reported in trials in haemodialysis, peritoneal dialysis and transplantation are extremely heterogeneous and are predominantly surrogate endpoints such as calcium, potassium and phosphate [9–13].

Across trials in patients with CKD who do not require dialysis or transplant, the outcomes reported in recent trials are also inconsistent with variability in the measures used. For example, kidney function has been reported across trials with different definitions and measures [8]. Three recent large trials in CKD (CKD-FIX [Controlled trial of slowing of Kidney Disease progression From the Inhibition of Xanthine oxidase], CREDENCE [Evaluation of the Effects of Canagliflozin on Renal and Cardiovascular Outcomes in Participants With Diabetic Nephropathy] and SONAR [Study Of Diabetic Nephropathy With Atrasentan] have reported and measured kidney function in different ways [14–16]. CKD-FIX measured kidney function as a single endpoint (change in eGFR) while CREDENCE and SONAR measured kidney function as a composite of end-stage kidney disease (dialysis, transplant or eGFR < 15 mL/min per 1·73 m^2^), doubling of serum creatinine and mortality. The time points for the definition of eGFR and duration of dialysis differed between CREDENCE and SONAR (30 days vs. 90 days). The variability in the outcomes and measures reported across trials in CKD and omission of patient-important outcomes makes it very difficult to compare the effect of interventions across trials and to make decisions based on outcomes that are meaningful to patients.

To improve consistency in reporting outcomes of critical importance to patients and health professionals across trials, the global Standardised Outcomes in Nephrology (SONG) initiative was launched in 2015 and has since established core outcome sets for trials in haemodialysis [17], transplant [18], peritoneal dialysis [19], polycystic kidney disease [20] and children and adolescents [15]. Core outcomes sets are an agreed upon standardised set of outcomes that should be measured and reported in all trials in a specific clinical field [21]. However, there is no core outcome set for trials in CKD (CKD stage 1–5, not requiring kidney replacement therapy). The SONG-CKD initiative addresses a gap by establishing a core outcome set that will be co-produced by patients, caregivers, clinicians and researchers. This core outcome set is likely to help improve the relevance of trials for informed decision-making among patients with CKD and their caregivers and health professionals.

## Methods/design

The SONG-CKD methodology is adapted from the processes used in SON G[22] and the Outcome Measures in Rheumatology (OMERACT )[23] and Core Outcome Measures in Effectiveness Trials (COMET) [21] initiatives which have been recognised as a comprehensive, transparent and robust approach for establishing core outcomes. SONG-CKD involves four phases: a systematic review, stakeholder key informant interviews, an online international Delphi survey, and consensus workshops.

### Phase 1: Systematic review of outcome domains reported in trials in CKD not requiring kidney replacement therapy

We will conduct a systematic review to identify and compare outcome domains and measures reported in randomised controlled trials (RCTs) of interventions for adults with CKD (not requiring kidney replacement therapy).

#### Search strategy

We will search the Cochrane Kidney and Transplant Register of Studies, MEDLINE, and Embase for all RCTs involving adult patients (aged 18 years and over) with CKD not receiving kidney replacement therapy. We will retrieve the full-text article of all RCTs included in the systematic review, and also search for all additional articles published from the same trial using key word searches in electronic databases (MEDLINE, Embase) and trial registries. No date or language restrictions will be applied.

#### Types of studies

RCTs in adult patients with CKD.

#### Types of interventions

Any intervention to manage patients with CKD will be eligible (including but not limited to surgical, pharmacological, psychosocial, diet and lifestyle).

#### Types of participants

Adult patients (aged 18 years or over) with CKD stages 1 to 5 (not requiring kidney replacement therapy). Studies which exclude patients with CKD or only enrol children (aged 18 years or below) with CKD will not be included. RCTs that only include patients receiving kidney replacement therapy in which the data from CKD patients (stages 1–5) are not reported separately, will be excluded.

#### Eligibility of studies

Two reviewers will independently assess all records obtained from the searches. The two reviewers will assess full texts of all RCTs independently, and any discrepancies will be evaluated with a third reviewer.

#### Data extraction

One reviewer will extract characteristics of all the trials including first author, date of publication, country/ies in which the trial was conducted, sample size, participant characteristics (range and mean age, gender, CKD stage, kidney disease diagnosis and measures of albuminuria/proteinuria), trial duration, name and type of intervention, registration number of the protocol, all outcomes as reported in the trial (including definitions, tools for measurement, thresholds, time points or time frames for measurement, change in level or percentage, scores) and the category of outcomes (primary or secondary). Two reviewers will cross check the data extraction.

#### Data analysis and presentation

The data will be entered into Microsoft Excel for data management, tabulation and analysis. All similar outcomes will be grouped into appropriate outcome domains, which will be reviewed and discussed by the SONG-CKD Steering Group. The outcome domains will be broadly classified as surrogate, clinical or patient-reported outcomes. We will identify the number of trials that reported each outcome domain. For each outcome domain, we will assess the number of different outcomes (including measures) and the number of trials that assessed each specific outcome. We will perform statistical analyses of the frequency of outcomes using the software package R version 3.2.3 (R Foundation for Statistical Computing, Vienna, Austria).

### Phase 2: Stakeholder interviews

Semi-structured interviews will be conducted to obtain and describe a range and depth of individual values, beliefs and attitudes from health professionals on establishing and implementing outcomes in trials in CKD [24]. Reporting will be based on the Consolidated Criteria for Reporting Qualitative Health Research (COREQ) [25].

#### Participants and recruitment

Health professionals including nephrologists, surgeons, nurses, allied health professionals (e.g. psychologists, social workers, dieticians), researchers, and policy-makers with experience in CKD will be eligible to participate in an interview. At least 50 participants will be recruited worldwide and selected from networks of the SONG-CKD Steering Group and Investigators. A purposive sampling approach will be used to enable a range of demographics, professional roles and experiences. We will recruit until data saturation, which we will define as when no new concepts or outcomes are being identified in three consecutive interviews. All participants will be asked to provide informed consent prior to the interview.

#### Data collection

The interview guide will incorporate findings from Phase 1 (systematic review) and interviews will be conducted face-to-face. However, if face-to-face interviews are not possible, we will use videoconference (Zoom video conferencing platform) or telephone interviews if preferred by the participants. Throughout the interview, participants will be asked to discuss their perspectives on (1) their experiences of managing patients with CKD and important aspects of treatment, (2) outcomes they believe are relevant for trials and why, (3) perceptions on core outcomes for trials in CKD, implementation strategies and any aspects that may be specific to the CKD population (not requiring kidney replacement therapy). The interview duration will be approximately 40 min and will be audio-recorded and transcribed.

#### Data analysis

We will use thematic analysis to identify and describe themes that reflect the perspectives, attitudes, beliefs and values from health professionals about core outcomes for research in patients with CKD. To ensure rigour, at least two investigators will be involved in coding the data analysis to develop descriptive and analytical themes (investigator triangulation). The preliminary results will also be reviewed by the participants (member checking) and the SONG-CKD Steering Group.

### Phase 3: International online Delphi consensus survey

An international online Delphi survey will be conducted to generate consensus on the outcomes that are most important to patients with CKD, caregivers and health professionals. The Delphi technique involves sequential surveys which are answered anonymously and it gives equal influence to all who participate [26, 27]. Applying the Delphi method in a rigorous and transparent manner can allow for consensus to be achieved on a complex topic and enable knowledge exchange and a new perspective on the topic among diverse participants. It is highly applicable for core outcome set development as it can reach and engage a wide range of stakeholders from multiple countries and has been used to obtain reliable consensus on core outcome sets across a range of health conditions [28–31].

#### Participants and recruitment

There is no minimum sample size requirement for a Delphi panel [32]. Based on previous SONG Delphi surveys, in which the sample size have ranged from 557 to 1,18 1[20, 33–36], our minimum target sample size will be 1000 respondents with at least 500 patients with CKD to ensure a balance between patients/caregivers and health professionals (nephrologists, surgeons, nurses, allied health professionals, policy makers (including regulators), researchers and representatives from industry); who have experience or interest in outcomes in CKD.

Purposive sampling and snowballing strategies (where participants can nominate or extend an invitation to other relevant stakeholder members to participate) will be used to ensure variation in demographics, clinical characteristics (patients), and professional roles (health professionals) as is feasible. Patients/caregivers will be recruited through participating hospital/university institutions, patient/consumer organisations, and the SONG Initiative database. Health professionals will be recruited via the networks of the investigators and professional nephrology societies. The survey will be administered in three languages: English, Spanish and French.

#### Data collection

##### Generating the list of outcomes

The Delphi survey will include outcome domains from the systematic review of outcomes reported in RCTs (Phase 1) and studies on patient priorities for outcomes in CKD [7, 37]. Of note, a recent study was conducted with 67 patients with CKD and their caregivers in Australia, the USA and the UK to identify and prioritise outcomes for trials in CKD [7]. The top eight ranked outcomes overall included: kidney function, kidney failure requiring kidney replacement therapy, fatigue, mortality, life participation, blood pressure, cognition and anxiety [7].

All outcomes will include a plain language definition. The survey will be reviewed by the SONG Executive and SONG-CKD Steering Group, which includes patients with CKD and their caregivers, and piloted with at least ten patients with CKD.

##### Survey administration

Informed consent will be obtained from all participants online and a standardised study information sheet will be provided in the introduction of the survey. The surveys will be completed online using Qualtrics Survey Software. Each participant will be given a unique identifier so their responses from Round 1 to 2 can be linked anonymously. At least three reminders will be sent to participants during the Delphi Rounds in an attempt to retain at least a 70% response rate in Round 2. Participants who complete the two rounds will receive a copy of the preliminary results.

##### Delphi Survey Round 1

Participants will be asked to rate the importance of outcomes for research in CKD using the GRADE 9-point Likert scale [38]. The visual scale used for each outcome will indicate ratings 1 to 3 as “limited importance”, 4 to 6 as “important, but not critical”, and 7 to 9 as “critical importance” and provide an option of “unsure”. Responses to the rating questions will be mandatory. Participants can provide comments in a free-text box under each outcome. The sequence of outcomes will be randomised to prevent ordering bias and at the end of the round, participants can suggest new outcomes. All new outcomes suggested by more than 10% of participants and which do not duplicate already presented outcomes, will be re-coded by at least two investigators and reviewed by the SONG-CKD Steering Group, then carried through to Round 2.

**Fig. 1:**
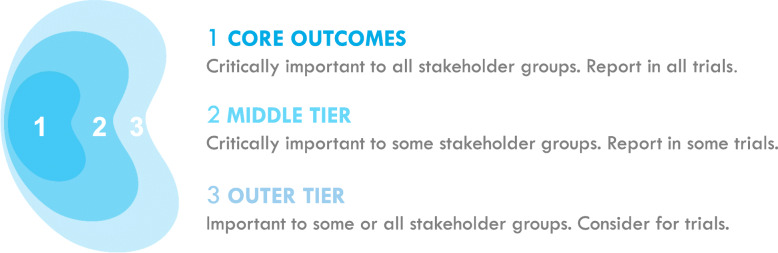
SONG conceptual schema of core outcomes

An outcome with a median and mean score of greater than 7 and greater than or equal to 70% of participants rating the outcome as critically important (7–9) in both patient/caregiver and health professional groups will be included in Round 2. Any outcomes excluded in subsequent rounds will be listed as important to some or all stakeholder groups to consider for trials (“outer tier” outcomes) or critically important to some stakeholder groups to report in some trials (“middle tier” outcomes) (see Fig. 1).

##### Delphi Survey Round 2

In Round 2 (the final round of the survey), participants will be presented with a column graph of the distribution of scores for each outcome for the following groups: (1) patients/caregivers, (2) health professionals and (3) all participants (with scores weighted evenly between groups). An explanation of how to read the graph with an example will be provided to assist participants in interpreting the graph. They will also see comments from patients/caregivers and health professionals and their own response from Round 1 highlighted on the Likert scale. Participants will re-rate each outcome and a free-text box will be provided for participants to explain reasons for their rating, or to provide responses to the comments.

In addition to the Likert scale, the final round of the survey will include a Best Worst Scale (BWS), a type of discrete choice experiment which allows participants to assess the relative importance of each outcome [39]. The BWS methodology has been used in previous SONG Delphi surveys [20, 34, 35] and has also been used to assess patient preferences in kidney transplantation [40].

#### Data analysis

For both rounds, we will summarise the distribution of scores and calculate the mean, median and proportion for the ratings of each outcome. A multinomial logistic regression model will be used to analyse the scores from the BWS and calculate the relative importance of each score based on a range of 1(least important) to 9 (most important). According to consensus criteria developed from previous SONG Delphi survey s[20, 33–36], inclusion in the core set requires that the outcome must have: a mean score greater than or equal to 7.5, a median score greater than or equal to 8 and at least 70% of participants scoring 7 to 9 (critically important) [41]. Based on previous studies, a core set includes three to five outcomes for feasibility. However, it is possible that more than five outcomes will meet the criteria for inclusion. Preliminary outcomes with the rationale and threshold for inclusion will be detailed in a plain language report and discussed at the consensus workshops for further input (Phase 4).

### Phase 4: Consensus workshops

Due to current COVID-19-related restrictions on the size of gatherings and international travel, we will convene online consensus workshops using the video conferencing platform Zoom. The workshops will be convened in English and Spanish with relevant stakeholders to comment and critique the potential set of core outcomes and to discuss strategies for the development of outcome measures and implementation. A member of the SONG-CKD Steering Group will chair each session. We will aim to have a maximum of 60 attendees; with at least 20 patients/caregivers. Health professionals with a range of clinical experience in CKD (nephrologists, surgeons, nursing and allied health professionals), expertise in research (epidemiology, clinical trials in CKD, registries, quality improvement), and leadership or advisory roles in major research and policy organisations (including regulators), and industry will be invited to attend.

Prior to the workshops, we will send participants a copy of the results from Phases 1-3 so they can reflect on the results and feel informed to contribute their opinions during the workshop. Each workshop will include three sessions:

Session 1: IntroductionWe will provide a brief introduction to the SONG-CKD initiative and present the details of the SONG-CKD process and results from Phases 1 to 3, and the proposed core outcome set.

Session 2: Breakout groupsParticipants will be allocated to approximately five virtual breakout groups with up to 10 participants in each group (including a facilitator and co-facilitator). Mixed stakeholder groups with at least 2 patients/caregivers will be convened to ensure representation and input from patients/caregivers. A trained facilitator will ask participants to discuss the identification and implementation of core outcomes and ensure a collaborative and comprehensive discussion. The facilitator will also seek to identify the context of participant responses and how socio-cultural factors may have informed the priorities of outcomes. All facilitators will attend a briefing session and will be provided with a question guide.

Session 3: Plenary discussionThe groups will move from their virtual breakout rooms and return to reconvene with the larger group and engage in a broader discussion moderated by the workshop chair. A member from each breakout group will present a brief summary of their discussion and the larger group will be invited to give their perspectives on the issues raised by other groups. The moderator will summarise the main points raised by the groups and highlight any similarities or differences.

## Discussion

SONG-CKD will establish a new set of critically important core outcome domains to be reported in all trials in CKD and involve patients, caregivers and health professionals in a transparent and equitable consensus process. Furthermore, the core outcome set will establish the basis for future work on identifying valid and feasible outcome measures to be reported for each outcome domain, such as suitable measures for kidney function (glomerular filtration rate and albuminuria).

We recognise there may be differences in priorities of outcomes dependant on the stage of CKD and presence of comorbidities. CKD may have a number of causes and be complicated by concurrent diseases such as diabetes and cardiovascular disease which contribute to progression of CKD and complications. We will conduct sub-group analyses according to stage of CKD, cause of CKD and current treatment. Outcomes relevant to these specific subgroups may be captured in the middle- and outer-tier outcomes (Fig. 1) and identified so trialists are aware they are important to use in trials which include these subgroups. We also recognise that socio-cultural factors are important in understanding the context of participant’s scores and rating of outcomes. We will conduct a sub-group analysis of the Delphi survey data by ethnicity, country and socio-economic status to better understand the context of these responses from the participants and further expand on this in the consensus workshop.

Strategies to effectively implement core outcome sets in trials in nephrology were discussed and established in a recent SONG implementation workshop involving 82 patients and caregivers and 76 health professionals and trialists [42]. A comprehensive approach including advocating for the inclusion of core outcome sets to trialists and other relevant stakeholders, demonstrating credibility, feasibility and usability and integrating core outcome sets into infrastructure such as registries and routine care were identified as key actions for overcoming barriers for the uptake of core outcome sets. The core outcomes may also be considered for other study designs, including observational studies.

Reporting outcomes that are critically important to patients and all appropriate stakeholder groups, will improve the quality and relevance of research evidence to inform decision-making and promote patient-centred care. Consistent reporting of relevant outcomes in clinical trials is expected to inform clinical practice and ultimately improve treatment of patients with CKD.

## Data Availability

Not applicable.

## Abbreviations

CKD: Chronic kidney disease
COMET: Core Outcome Measures Effectiveness Trials
FDA: Food and Drug Administration
OMERACT: Outcome Measures Rheumatoid Arthritis Clinical Trials
RCT: Randomised controlled trial
SONG-CKD: Standardised Outcomes in Nephrology – Chronic Kidney Disease
